# Supplementary material for: Artificially controlled nanoscale chemical reduction in VO2 through electron beam illumination
Source: Nat Commun. 2023 Jul 7;14:4012. doi: 10.1038/s41467-023-39812-8 (PMC10329014; doi:10.1038/s41467-023-39812-8)
Supplement: Supplementary file 1 — Supplementary Information [file 41467_2023_39812_MOESM1_ESM.pdf]

Supplementary materials for

Artificially controlled nanoscale chemical reduction in VO_2 through electron beam illumination

Yang Zhang, Yupu Wang, Yongshun Wu, Xinyu Shu, Fan Zhang, Huining Peng, Shengchun Shen, Naoki Ogawa, Junyi Zhu and Pu Yu

This PDF includes

Supplementary text

Reference

Figure S1-S18

Table S1-S3.

1. Mechanism of e-beam induced phase transformation from VO_2 to V_2O_3

After excluding the role of knock-on effect and heating effect through other experiments, we now turn our focus to the radiolysis effect. In solid matters, the radiolytic process can lead to the formation of stable defects within the bulk via nonradiative relaxation of excitation. The decay of electronic excitations breaks the chemical bonds, ultimately resulting in the formation of defects [1, 2]. The radiolysis plays a major role to describe the defect formation and amorphization in halides and zeolites [3-4]. While, in oxides, the radiolysis typically produces the formation of Frenkel-type defects, consisting of O-O peroxyl species and O vacancies, which contribute to the amorphization of crystalline SiO_2 [5]. Interestingly, in transition metal oxides, the radiolytic process can also trigger oxygen desorption from the surfaces due to the transition from O^{2-} to O^0 or O^+ ions [6, 7]. This desorption process will subsequently lead to the reduction of the metal ions and a corresponding structural change at the surface. This mechanism primarily applies in high valent ionic materials, such as TiO_2, V_2O_5 and WO_3, resulting in the formation of monoxide phase at the surface [8-10].

Therefore, we attribute the main mechanism of e-beam induced phase transformation from VO_2 to V_2O_3 as following. Under moderate illumination, an internal electric field is formed by the positive background at the surface. Additionally, the radiolytic effect can induce the oxygen desorption from the surface, resulting in respective reduced phases through the formation of surface oxygen vacancies, and this would create a gradient chemical potential for oxygen ions between the surface and sample bulk [11, 12]. We note that these two mechanisms contribute cooperatively to facilitate the diffusion of oxygen vacancies into the bulk, ultimately driving the phase transformation.

2. Theoretical consideration of VO_2 and V_2O_3, as well as their corresponding surfaces

We conducted the electron counting model [13–16] (ECM) to accelerate the searching process for the most stable surface and interface configurations. To start, we assumed that the valence of the V atom in VO_2 is +4, and the V and O atoms are six-fold coordinated [17-18]. By inspecting the coordination, it can be determined that each V atom is connected to six O atoms, and each O atom is connected to three V atoms. Thus, each V atom contributes four electrons to its six coordinations, that is with 2/3 electrons for each coordination. Similarly, each O atom denotes six electrons, with one electron for each coordination. Because of the octet rule, each V-O bond should contain 8/6 (4/3) electrons. Therefore, each V atom has 1/3 electron excess for each coordination. Additionally, each O atom is bonded with three V atoms, and three coordinations for each O atom are not fully filled. Therefore, each O atom has 1/3 electron missing

for each coordination. As a result, for each V and O atom, there are two electrons excess (1/3×6) and one electron lacking (1/3×3), respectively. In summary, for each formula, 1×(+2) (V) + 2×(-1) (O) = 0. Therefore, if one V-O bond is broken on the surface, each V and O atom has 1/3 electron excess and lacking, respectively.

Based on this counting, we constructed 6 configurations of VO_2 surfaces and listed them in **Fig. S15**. Following the strategy proposed in reference [19], we counted the electrons on each layer (or surface) of the slab, with symmetrical top and bottom surfaces. Therefore, the total ECM of the slab can be obtained by summing the electrons on all layers and surfaces. For the a-facet terminated VO_2 slab, we found that there are five types, as shown in **Fig. S15** from type 1 to type 5. Among these types, the type 2, 4 and 5, satisfy the ECM automatically, while the type 1 and 3 do not. We also found that the most stable surface termination is type 5 and adopted this slab in the following calculations. For the c-facet, there is only one type of surface termination (right panel in **Fig. S15**) that satisfy the ECM. Therefore, we adopted this configuration in the following calculation.

A similar treatment was applied to V_2O_3. Here, we assumed the chemical valence of V is +3, and the O atom is four-coordinated. Therefore, for each V atom, there is one electron excess, and for each O atom, there are two partially filled coordinations, with 2/3 electrons missing. In summary, 2×(+1) (V) + 3×(-2/3) (O) = 0. Therefore, if one V-O bond is broken on the surface, each V and O atom has 1/3 excess and lacking, respectively.

3. Electron counting model and local distortions of VO_2/V_2O_3 interfaces

We constructed the interfacial crystalline structures based on the TEM results and searched for the most stable configurations based on the ECM [20]. In the ECM, a bond breaking, that the coordination deviates from the typical coordination near the interface region, results in 1/3 electron per each coordination excess for the V atom or lacking for the O atom, respectively. Here, the typical V atoms are six-fold coordinated for both VO_2 and V_2O_3 systems while the O atoms in the VO_2 and V_2O_3 are three-fold and four-fold coordinated, respectively. By counting all the electron supplies and demands of each coordination, we found three interface slabs (**Fig. S18**) satisfying the ECM after DFT relaxation.

However, large distortions occur near the interface region in interfaces I and II after DFT relaxation, as shown in **Fig. S18**. In interface I, the V atoms change from six-fold coordinated to five- or seven-fold coordinated, and some O atoms have one-fold coordination deviated from the VO_2 and V_2O_3 bulk. In interface II, some V atoms become four- and five-coordinated and many O atoms are also deviated from

the coordination of bulk. However, in interface III, the coordinations of V atoms are the same as that in bulk and only six O atom have one-fold coordination deviated from the bulk coordination. This indicates that the interface III is energetically more stable with less distortions. The exact numbers of deviated coordinations for each interface are summarized in **Table S3**.

4. The phase diagram of VO_2 and coexistent VO_2/V_2O_3

a) The phase diagram in the surface formation energy calculations

To find out the suitable chemical potential range for the calculations, we computed the phase diagram of VO_2 and VO_2/V_2O_3 and located the coexistence region based on calculated formation energies of elemental phases and experimental values of all compounds, in which the algorithm of fitted elemental-phase reference energy (FERE) was adopted in phase stability analysis to improve the accuracy [21, 22]. To form the stable crystal of VO_2, $\Delta\mu_{\mathrm{V}} + 2\Delta\mu_{\mathrm{O}} = \Delta H_f(\mathrm{VO_2}) = -7.35\ \mathrm{eV}$, where n_i, $\Delta\mu_i$ and $\Delta H_f(\mathrm{X})$ represent the number of elements and chemical potential in reference to the calculated bulk value of each element i, and the formation energy of compound X.

We considered five secondary phases, VO, V_2O_3, V_2O_5, and elementary substances (V and O_2) for the phase diagram calculations. To avoid the precipitation of these five secondary phases, the following relations should be satisfied,

$$\Delta\mu_{\mathrm{V}} + \Delta\mu_{\mathrm{O}} < \Delta H_f(\mathrm{VO}) = -4.45\ \mathrm{eV},$$

$$2\Delta\mu_{\mathrm{V}} + 3\Delta\mu_{\mathrm{O}} < \Delta H_f(\mathrm{V_2O_3}) = -12.58\ \mathrm{eV},$$

$$2\Delta\mu_{\mathrm{V}} + 5\Delta\mu_{\mathrm{O}} < \Delta H_f(\mathrm{V_2O_5}) = -15.97\ \mathrm{eV},$$

$$\Delta\mu_{\mathrm{V}} < 0, \Delta\mu_{\mathrm{O}} < 0.$$

b) The phase diagram in the interface formation energy calculations

As for the coexistent VO_2/V_2O_3 phase diagram, the relations become as follows,

$$\Delta\mu_{\mathrm{V}} + \Delta\mu_{\mathrm{O}} < \Delta H_f(\mathrm{VO}) = -4.45\ \mathrm{eV},$$

$$2\Delta\mu_{\mathrm{V}} + 3\Delta\mu_{\mathrm{O}} > \Delta H_f(\mathrm{V_2O_3}) = -12.58\ \mathrm{eV},$$

$$\Delta\mu_{\mathrm{V}} + 2\Delta\mu_{\mathrm{O}} > \Delta H_f(\mathrm{VO_2}) = -7.35\ \mathrm{eV}$$

$$2\Delta\mu_{\mathrm{V}} + 5\Delta\mu_{\mathrm{O}} < \Delta H_f(\mathrm{V_2O_5}) = -15.97\ \mathrm{eV},$$

$$\Delta\mu_{\mathrm{V}} < 0, \Delta\mu_{\mathrm{O}} < 0.$$

5. Surface and interface formation energy

In the slab method, the formation energy of the surface σ_s with the same top and bottom surface

termination is defined as $\sigma_s = \frac{E_{slab} - \sum_i n_i \mu_i}{2A_{surface}}$, where E_{slab}, μ_i and $A_{surface}$ are the energy of the slab with the surface, the chemical potential of elements species *i*, and the area of the surface, respectively. Similarly, the formation energy of interface σ_i is given by $\sigma_i = \frac{E_{supercell} - \sum_i n_i \mu_i}{2A_{interface}}$, where $E_{supercell}$ is the total energy of the supercell with the interface and $A_{interface}$ denotes the area of the interface.

6. Construction of simulation cells of interface and corresponding lattice mismatch

As it is impracticable to exhaust all possible interfaces due to the limited computer resource, we built up the characteristic interfaces as inspired by the TEM results. To obtain the stable configurations of both VO_2 and V_2O_3 parts, the lattice constants of simulated interface cells are adopted by the average values of VO_2 and V_2O_3. Even if two phases can become commensurate on large scale, there still could have some dangling bonds leading to interface reconstruction.

For interfaces I-III, we adopted the simulation cell size as a supercell of 2×3, 2×2 and 3×2 periods of VO_2 primitive cell and 1×1, 1×2 and 1×2 periods of V_2O_3 primitive cell, respectively. The lattice mismatch between these two phases is defined as $M = \frac{d(\mathrm{V_2O_3}) - d(\mathrm{VO_2})}{d(\mathrm{VO_2})}$, where $d(\mathrm{VO_2})$ and $d(\mathrm{V_2O_3})$ are the lattice constant of VO_2 and V_2O_3 supercells along in-plane direction of the interface, as shown in **Table S1.**

Reference

[1] M. N. Kabler, et al. Vacancy-interstitial pair production via electron-hole recombination in halide crystals, *Phys. Rev. B* **60** 181948, 1978

[2] L. W. Hobbs, et al. Radiolysis and defect structure in electron-irradiated α-quartz, *J. Phys. Colloque* **41** 237, 1980

[3] W. A. Sibley, et al. Radiation damage processes in insulating materials, *Nucl. Instrum. Methods Phys. Res.* B **1** 419, 1984

[4] R. L. Salamanca, et al. Electron beam-induced damage and structure of SbCl5-graphite intercalation compounds, *Phys. Rev.* B **33** 2738–48, 1986

[5] L. W. Hobbs, et al. The role of topology and geometry in the irradiation-induced amorphization of network structures, *J. Non-Cryst. Solids* **182** 27–39, 1995

[6] R. D. Ramsier, et al. Electron-stimulated desorption: principles and applications, *Surf. Sci. Rep.* **12** 246–376, 1991

[7] M. L. Knotek, et al. Ion desorption by Core-Hole Auger Decay, *Phys. Rev. Lett*. **40** 964, 1978

[8] A. K. Petford, et al. Atomic imaging of oxygen desorption from WO_3 surface, *Surf. Sci.* **172** 496–508, 1986

[9] H. J. Fan, et al. Phase transitions in V_2O_5 in a high-resolution electron microscope, *Ultramicroscopy* **31** 357–64, 1989

[10] D. J. Smith, et al. The Electron-beam-induced reduction of transition metal oxide surfaces to metallic lower oxides, *Ultramicroscopy* **23** 299–304, 1987

[11] Y. Park, et al. Anionic flow valve across oxide heterointerfaces by remote electron doping, *Nano Lett.* **22** 9306-9312, 2022

[12] Y. Park, et al. Directional ionic transport across the oxide interface enables low-temperature epitaxy of rutile TiO_2, *Nat. Comm.* **22** 9306-9312, 2022

[13] J. B. Goodenough, The Two Components of the Crystallographic Transition in VO_2, *J. Solid State Chem.* **3**, 490 (1971).

[14] J. Jeong, et al. Suppression of Metal-Insulator Transition in VO_2 by Electric Field-Induced Oxygen Vacancy Formation, *Science* **339**, 1402 (2013).

[15] B. Xiao, et al. Testing the Jacob's Ladder of Density Functionals for Electronic Structure and Magnetism of Rutile VO_2, *Phys. Rev. B* **90**, 085134 (2014).

[16] H. Yoon, et al. Reversible Phase Modulation and Hydrogen Storage in Multivalent VO_2 Epitaxial Thin Films, *Nat. Mater.* **15**, 1113 (2016).

[17] S.-C. Tsang, et al. Towards Understanding the Special Stability of $SrCoO_{2.5}$ and $HSrCoO_{2.5}$, *Phys. Rev. Mater.* **3**, 2 (2019).

[18] Y. Wang, et al. Surface Stability Analysis with H Adsorption Affected the Magnetic Fluctuation of Brownmillerite $SrCoO_{2.5}$ Based on the Electron Counting Model by Layers, *J. Phys. Chem. C* **126**, 12251 (2022).

[19] J. C. Woicik, et al. Chemical Bonding and Many-Body Effects in Site-Specific x-Ray Photoelectron Spectra of Corundum V_2O_3, *Phys. Rev. B* **76**, 165101 (2007).

[20] J. Zhang, et al. Hydrogen-Surfactant-Assisted Coherent Growth of GaN on ZnO Substrate, *Phys. Rev. Mater.* **2**, 013403 (2018).

[21] M. W. Chase, et al. JANAF Thermochemical Tables, 1982 Supplement, *J. Phys. Chem. Ref. Data* **11**, 3 (1982).

[22] V. Stevanović, et al. Correcting Density Functional Theory for Accurate Predictions of Compound Enthalpies of Formation: Fitted Elemental-Phase Reference Energies, *Phys. Rev. B* **85**, 11 (2012).

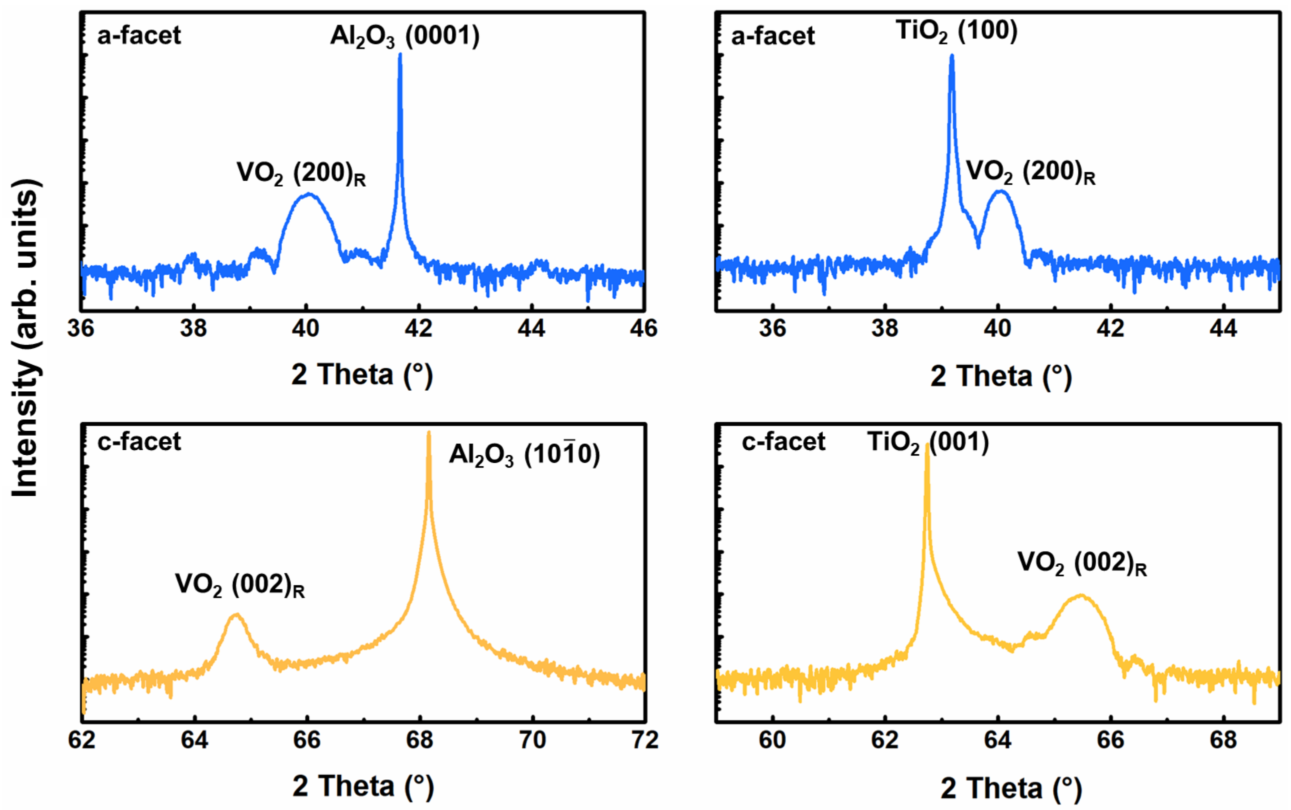

Figure S1. XRD measurements of a-facet (top panels) and c-facet (bottom panels) VO_2 thin films deposited on corresponding Al_2O_3 and TiO_2 substrates.

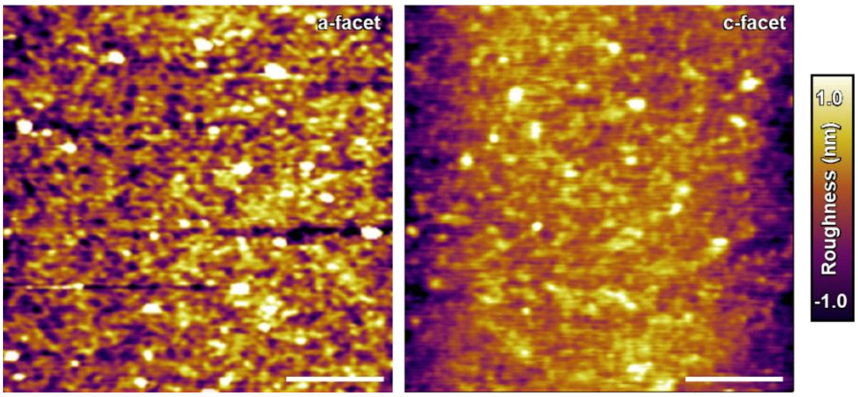

Figure S2. Atomic force microscope images of pristine a- and c-facet VO_2 thin films. The scale bar is 1μm.

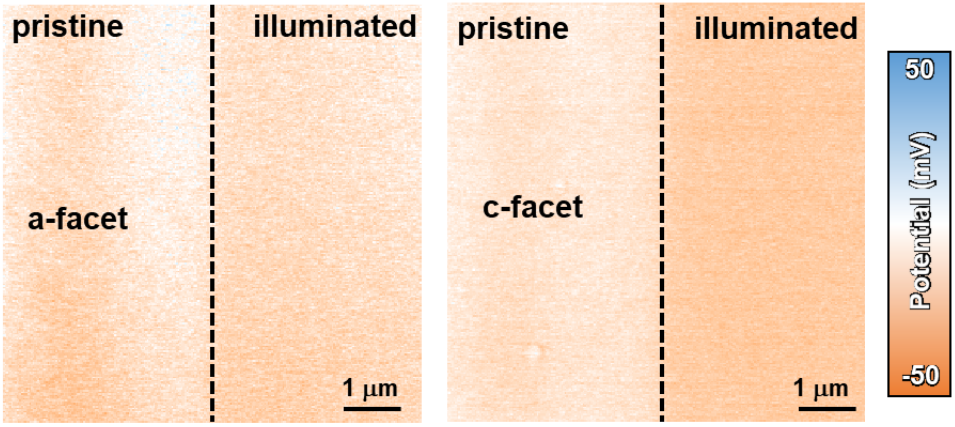

Figure S3. Surface potential maps of a-facet and c-facet VO_2 samples before and after e-beam illumination. The negligibly small difference of surface potentials excludes the scenario of e-beam induced charge injection.

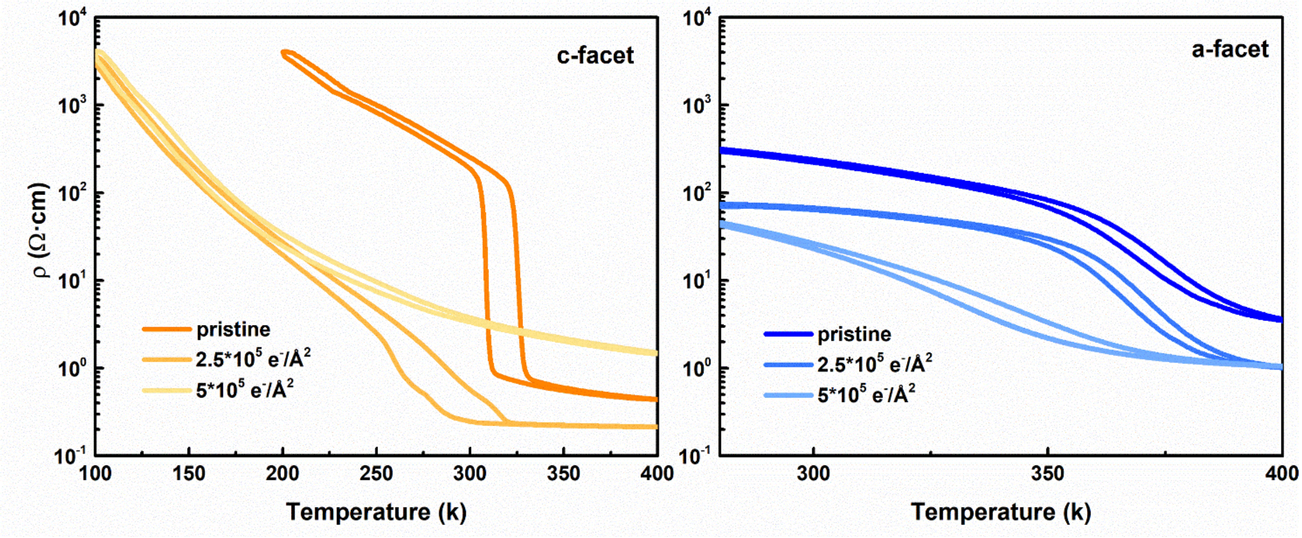

Figure S4. Temperature-dependent resistivity measurements via electron beam illumination at different electron doses. Left panel: R-T results for c-facet sample. Under the electron dose of 5×10^5 e/nm^2 illumination, the result shows signatures of both VO_2 (at high-temperature) and V_2O_3 (at low-temperature), indicating the formation of an intermediate state composed by both VO_2 and V_2O_3 phases. Right panel: R-T results for a-facet sample. Under the electron beam illumination of the same doses, the sample shows only a reduced IMT transition temperature, which can be attributed to the formation of small amount of (disordered) oxygen vacancies.

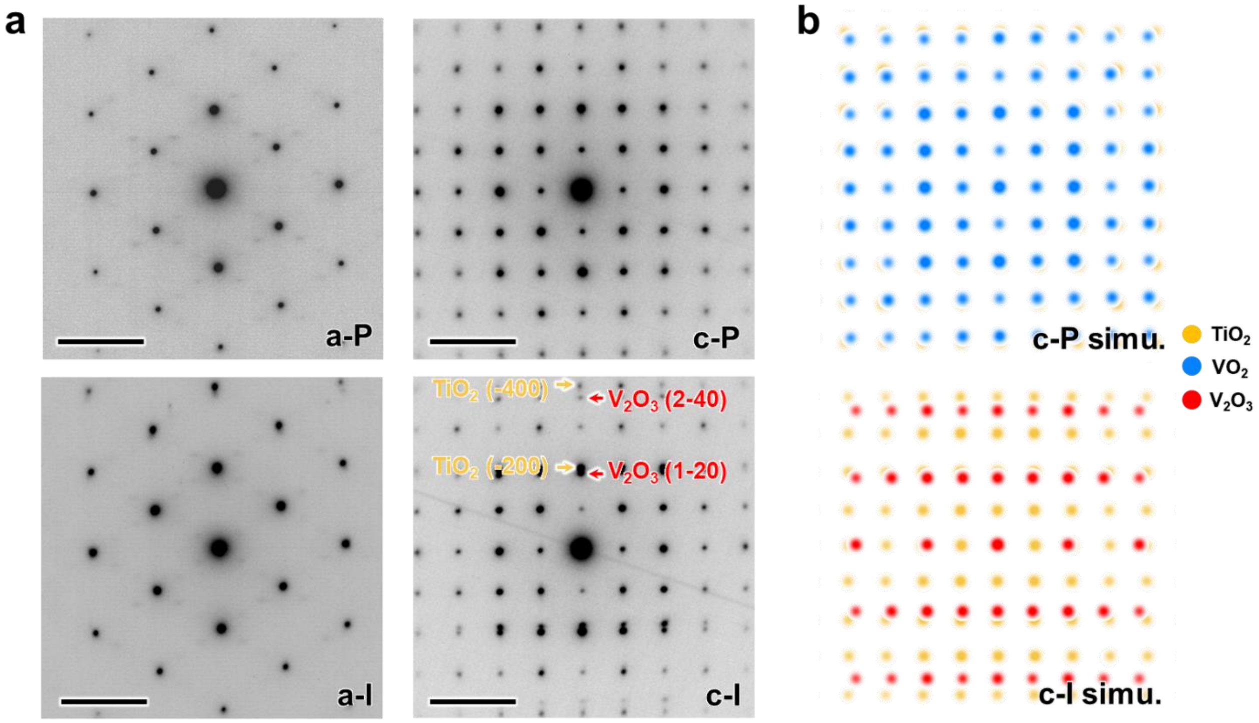

Figure S5. Diffraction patterns collected from pristine and illuminated regions. (**a**) Left panel: diffraction patterns collected from a-facet pristine VO_2 (a-P) and sample after e-beam illumination (a-I). Right panel: diffraction patterns collected from c-facet pristine VO_2 (c-P) and sample after e-beam illumination (c-I). (b) Simulated diffraction patterns of c-fact VO_2 and V_2O_3 samples superimposed with TiO_2. The simulated patterns show an excellent agreement with the experimental images.

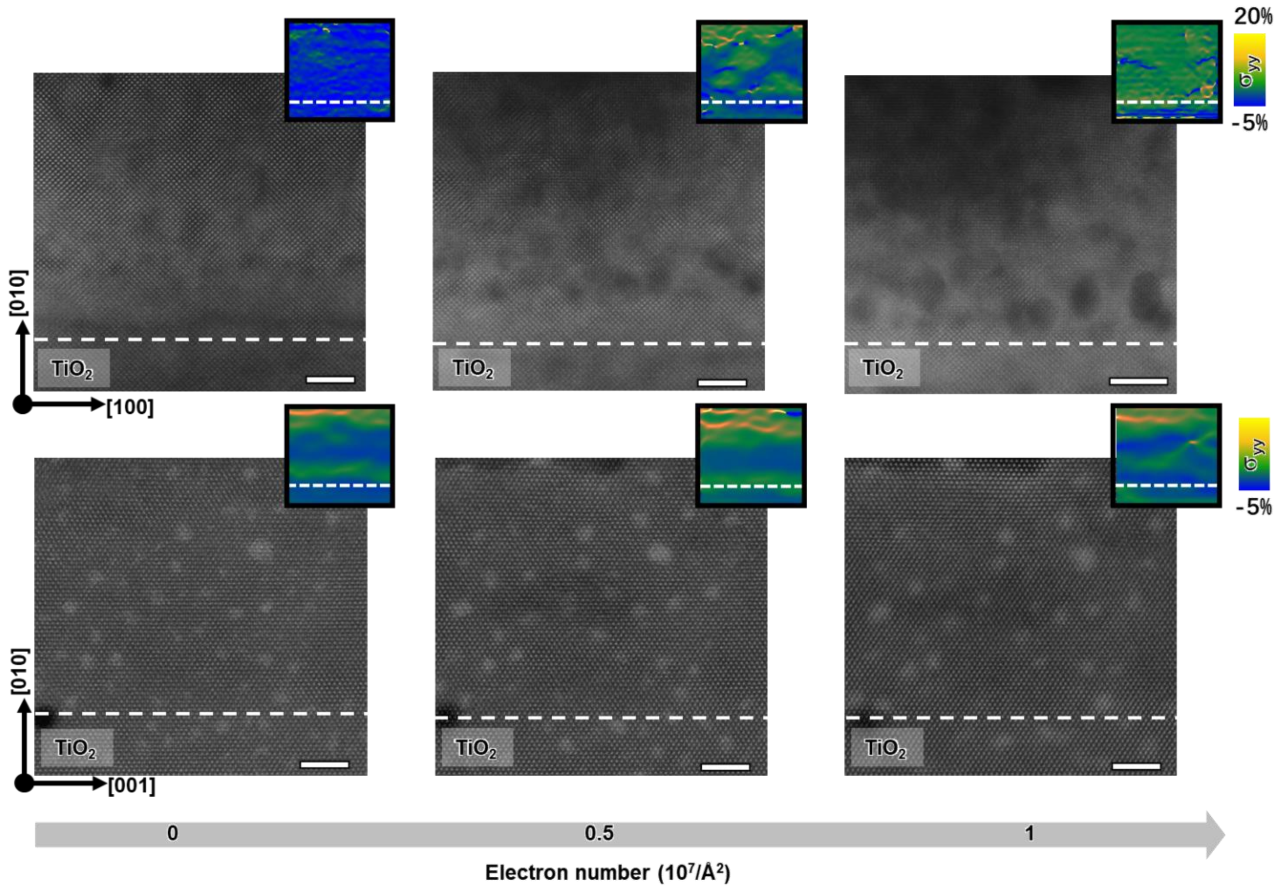

Figure S6. Upper panel: Evolution of c-facet VO_2 sample under continuous e-beam illumination. Lower panel: Evolution of a-facet VO_2 under continuous e-beam illumination. The inset shows corresponding GPA results at different illuminating conditions. The thickness of illuminating area was determined by the zero-loss of EELS, which is about 80 nm for both samples. The scale bar is 2nm.

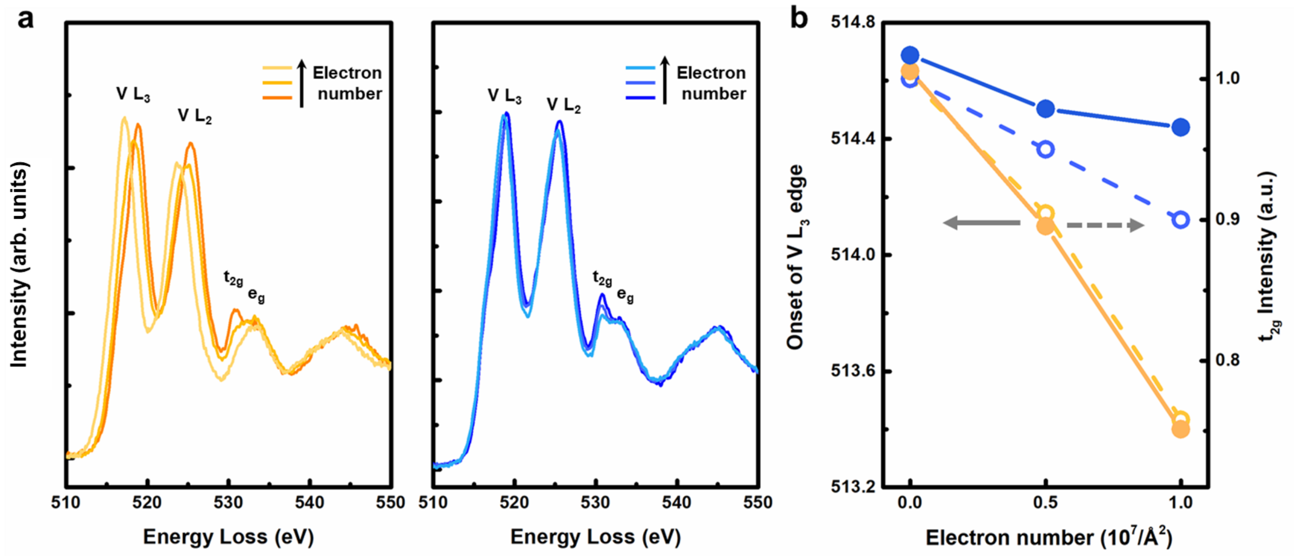

Figure S7. Evolution of electronic structures under continuous e-beam illumination. (**a**) Core-loss signals at vanadium *L*-edges and oxygen *K*-edges collected from c-facet and a-facet VO_2 samples under different illuminating conditions. (**b**) Variation of t_{2g} peak intensities at oxygen *K*-edges and positions of vanadium L_3-edge under different illuminating conditions. Results shown in (**a**) and (**b**) confirm the creation of oxygen vacancy for c-facet VO_2 sample and subsequent phase transformation into V_2O_3, while the a-facet VO_2 sample maintains its pristine structure with the formation of only small amount (~3%) oxygen vacancies.

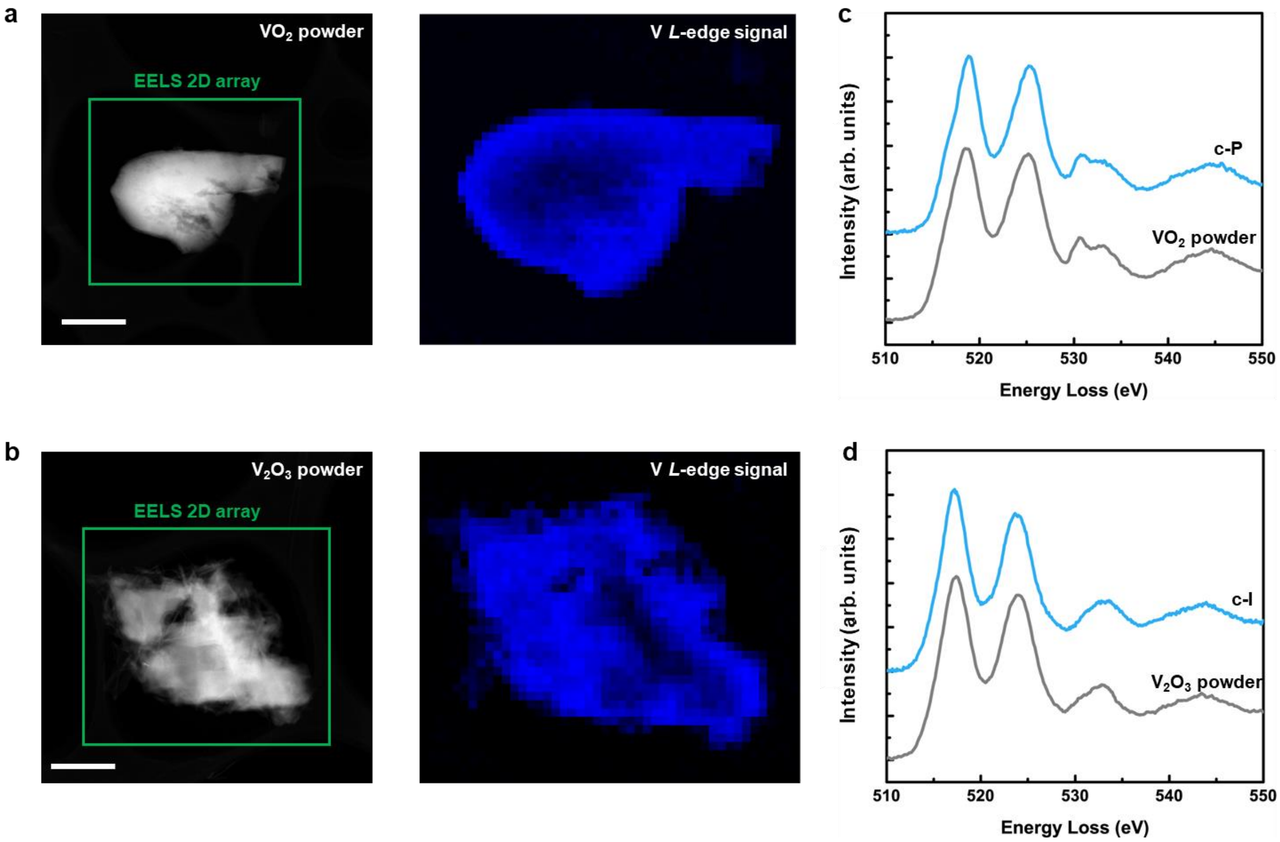

Figure S8. Comparison of EELS spectra between powders and c-facet thin films. (**a, b**) STEM-HAADF images (left panel) and EELS spectral images of (a) VO_2 and (b) V_2O_3 powders (right panel). (**c, d**) EELS spectra collected from powders (gray curves) and c-facet VO_2 before and after e-beam illumination (blue curves).

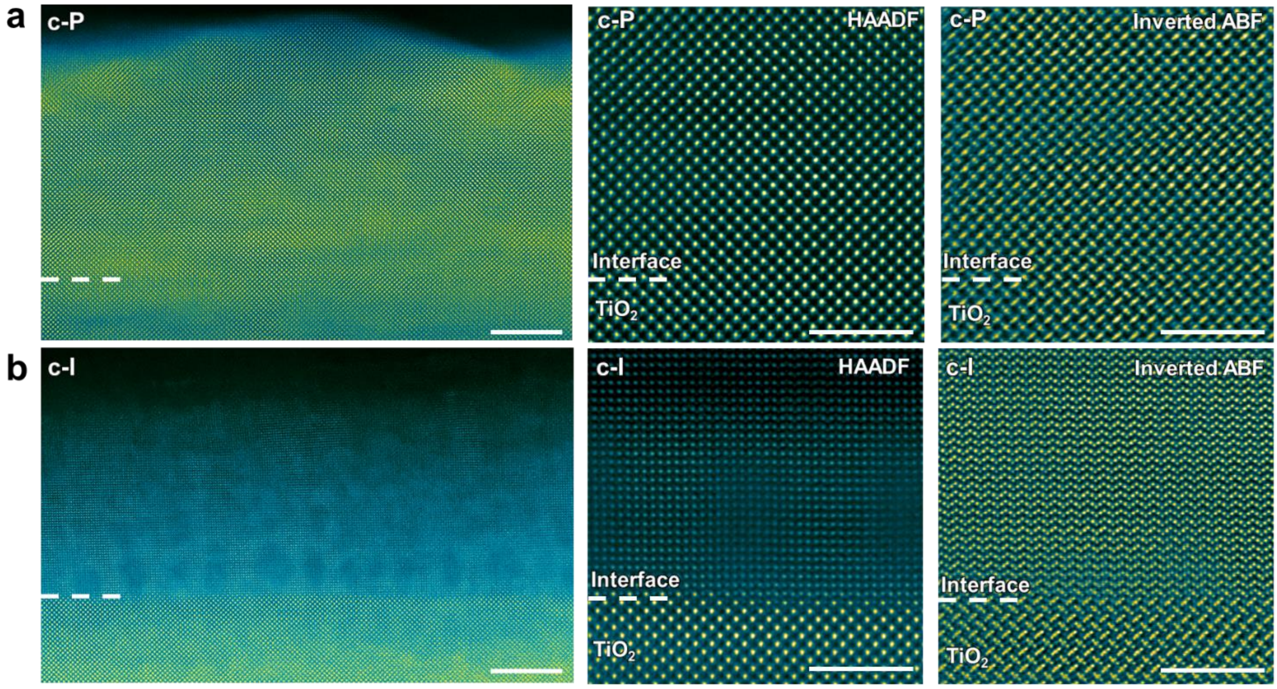

Figure S9. STEM images at (**a**) pristine and (**b**) illuminated regions for c-facet sample. Left panel: Low-magnified HAADF images; Middle panel: HAADF images; Right Panel: Inverted ABF images. The scale bars in low-magnified and high-magnified images are 5 nm and 2 nm, respectively.

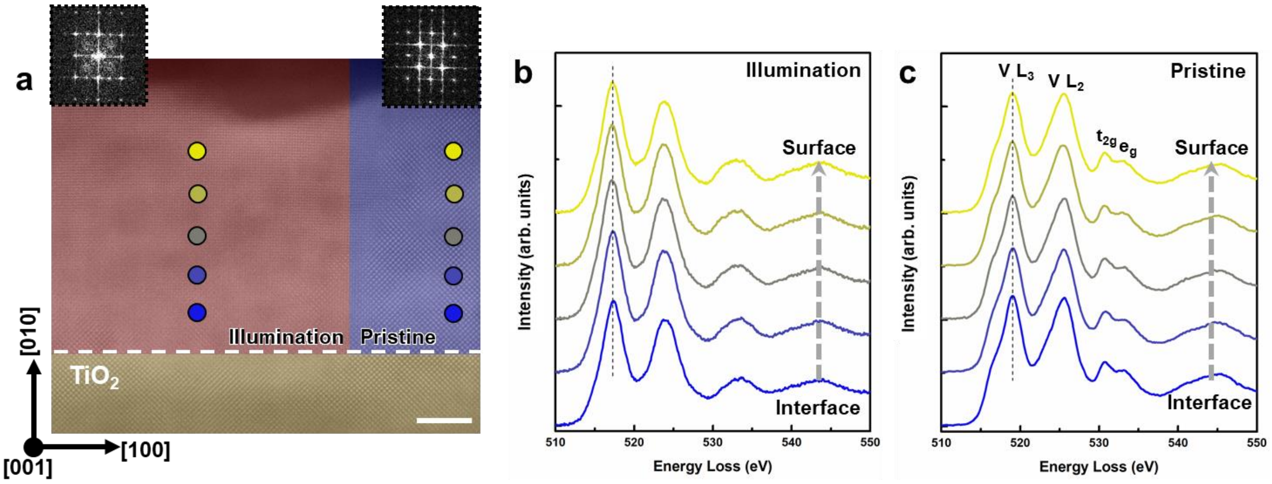

Figure S10. Depth-dependent EELS measurements of pristine and illuminated c-facet VO_2 thin films. (**a**) STEM-HAADF images collected at the boundary between illuminated and pristine regions. Corresponding FFT results are shown in the inset. The circles show the position with EELS signal extraction. The scale bar is 5 nm. (**b, c**) Depth-dependent EELS measurements extracted from illuminated and pristine regions. The black dotted lines serve as a guideline.

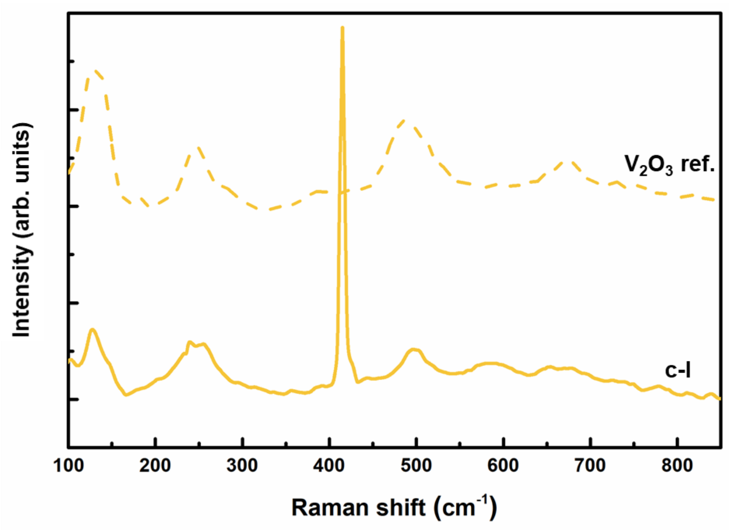

Figure S11. Comparison of Raman spectra taken at both illuminated c-facet sample and V_2O_3 powder.

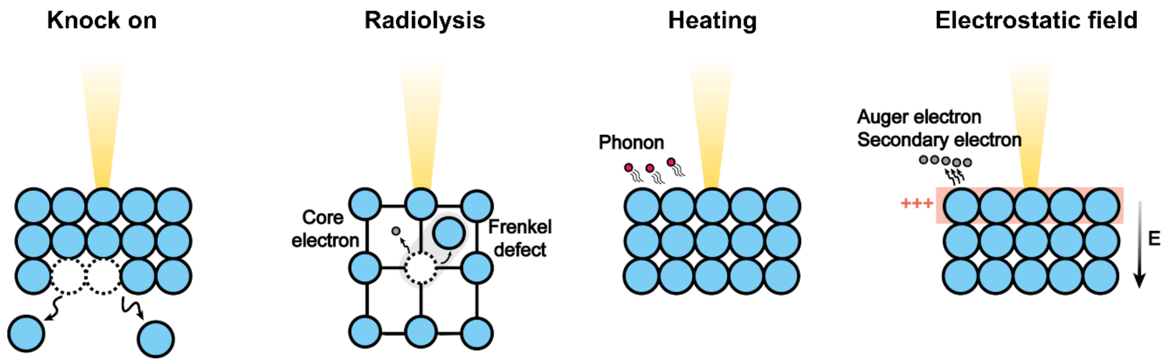

Figure S12. Summary of possible interactions between e-beam and materials. The knock-on effect is the direct collision between the high energy e-beam penetrating through the materials and the heavy ions. The radiolysis is induced by the loss of core-electron and the consequent formation of Frenkel defects. The heating effect is introduced by the excitation of phonons through e-beam illumination. The electrostatic field is caused by a positively charged sample due to the escaping Auger and secondary electrons.

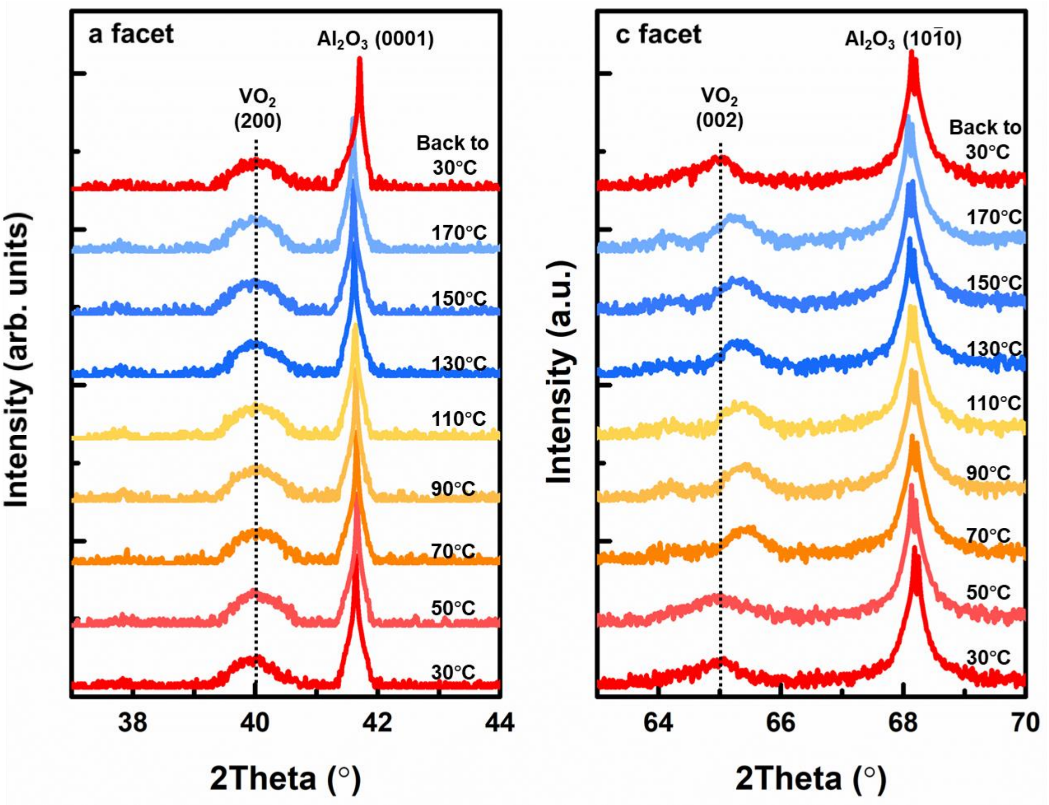

Figure S13. Temperature dependent *in-situ* XRD measurements. The warming process was conducted at ~1 Pa atmosphere. The a-facet sample shows a rather robust peak position with the temperature up to 170 °C through the thermal heating, which should be attributed to the closely matched lattice [100] constants between monoclinic and rutile phases in VO_2. While, for c-facet sample, the characteristic peak shifts into higher angle at 70 °C due to structural transformation, and then remains robust with the temperature up to 170 °C. The fact that the XRD peak recovers back to its pristine position again when temperature cools down to room-temperature (highlighted by black dotted lines) indicates that the VO_2 phase remains unchanged during the thermal heating. Considering the rather small dose of electron used, we expect the sample temperature is much less than 170 °C, and therefore the thermal heating effect can be excluded.

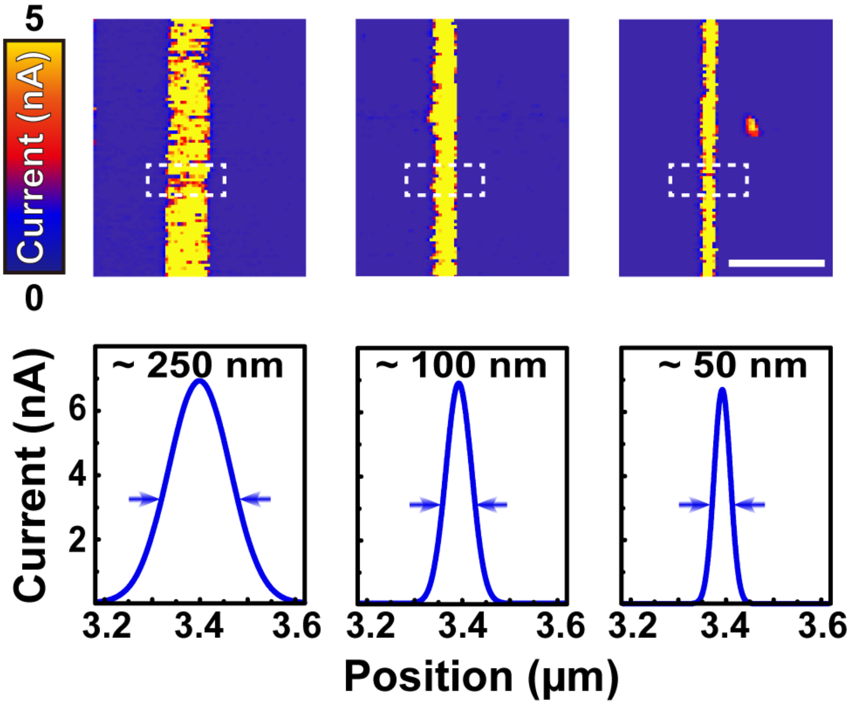

Figure S14. Estimation of featured size. Upper panel: Enlargement of current maps for a series of stripes shown in **Fig. 3**. The scale bar is 500 nm. Lower panel: Fitting of current profiles in (a) using the Gaussian functions. The feature sizes are estimated by the full width at half-maximum of the current profiles.

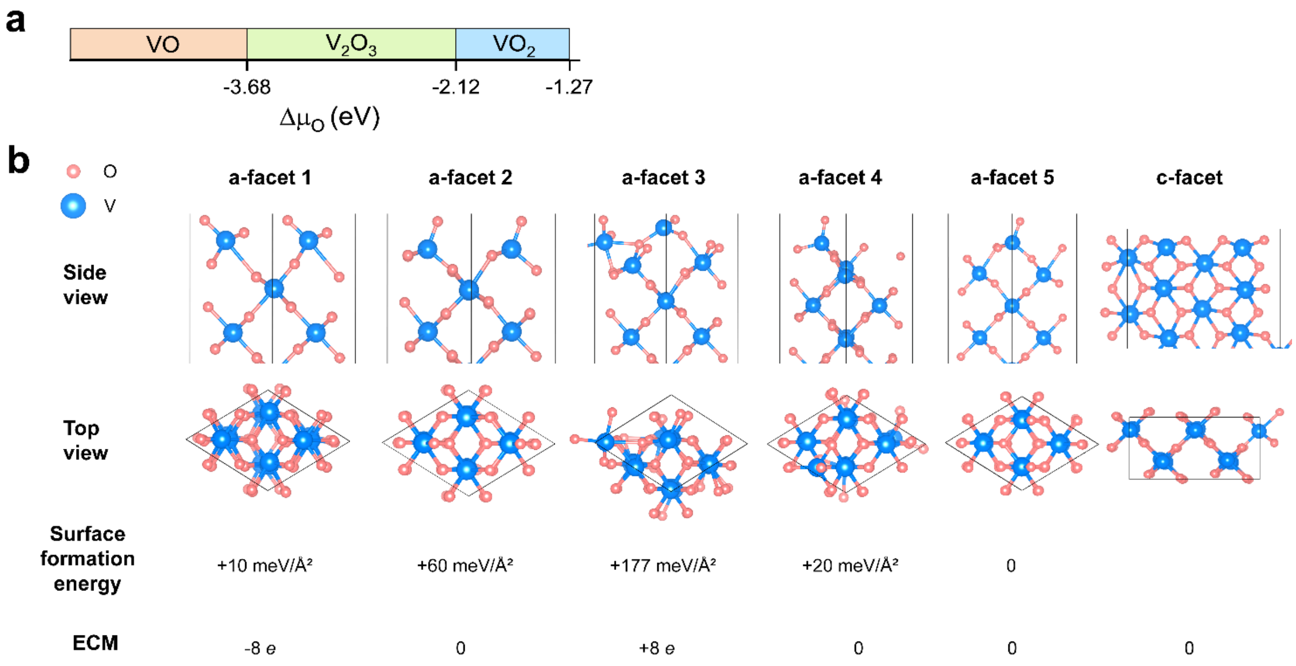

Figure S15. Phase diagram and surface stability of VO_2. (**a**) Phase diagram of VO_2. (**b**) Considered possible surface terminations for a-fact and c-fact samples. The ECM represent the total electrons of slab demands (-) or supplies (+).

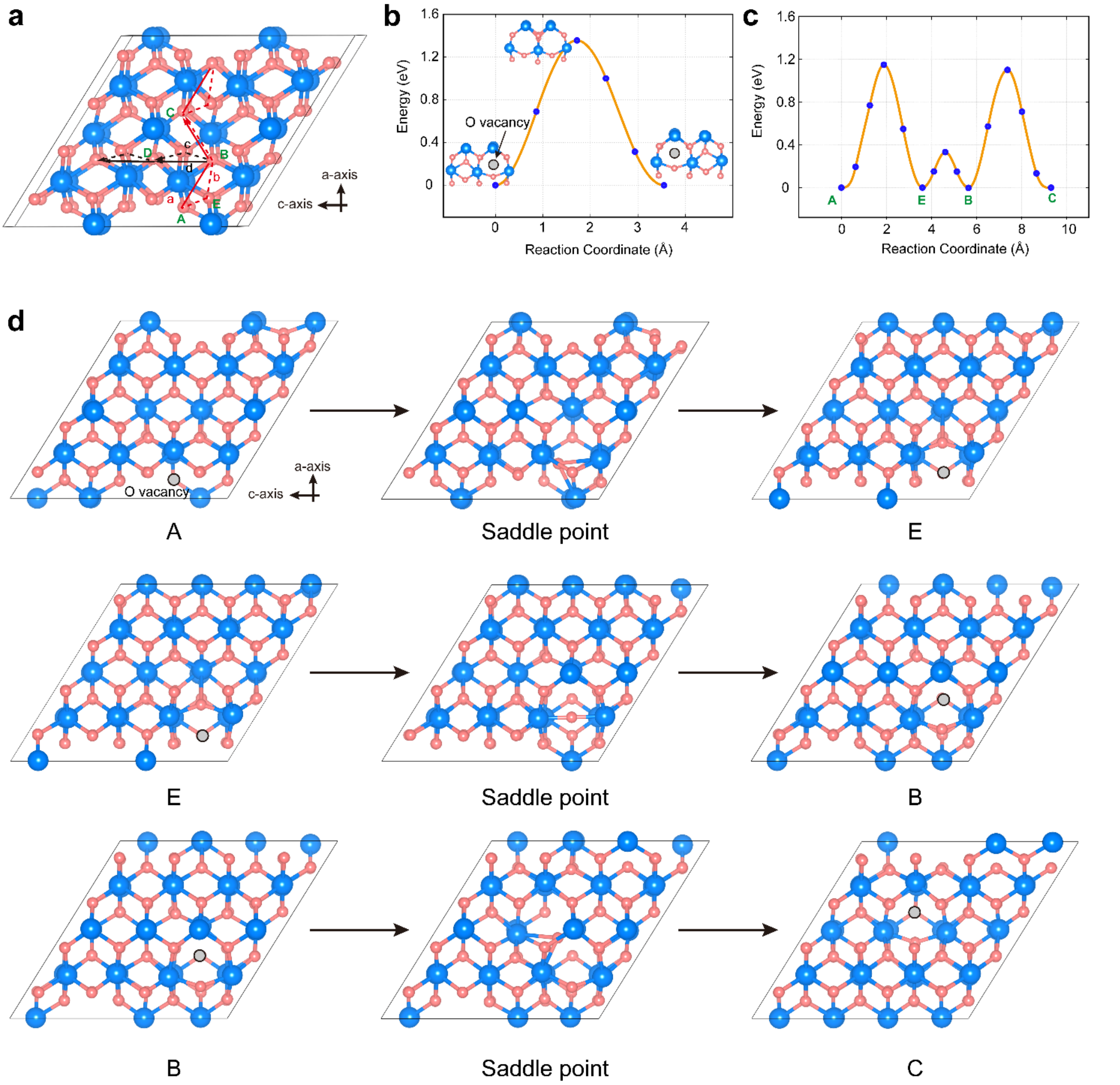

Figure S16. Calculated diffusion pathways of oxygen vacancy in bulk VO_2 along a- and c-axes. (**a**) Schematic illustrations of possible diffusion pathways for oxygen vacancies. Paths a-d are four possible diffusion pathways for oxygen vacancies between neighbored sites, and the other pathways can be reproduced through the combination of these paths. Paths a and c are the pathways with the lowest diffusion barriers and are discussed in the main text. (**b**) Energy profile for path d along c-axis. The inserts show the local crystalline structures around the oxygen vacancies. (**c**) Energy profile for path b along a-axis. The diffusion process can be divided into three subsequent steps. (**d**) Schematic illustrations of local crystalline structures during the diffusion with the steps labeled in (c).

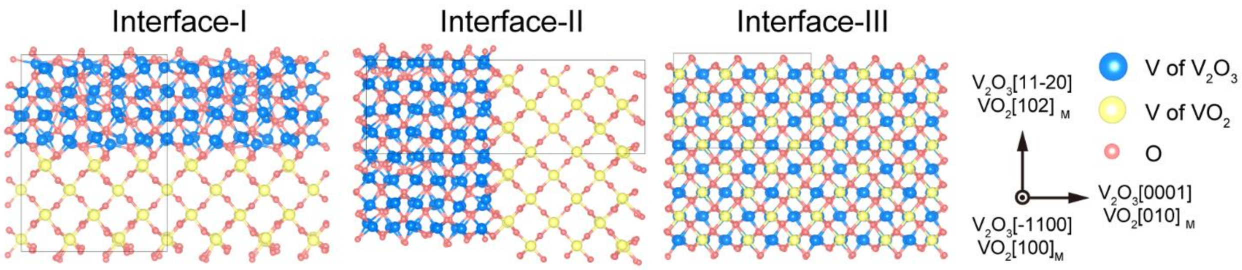

Figure S17. Crystalline structures between VO_2 and V_2O_3 constructed for the DFT calculations.

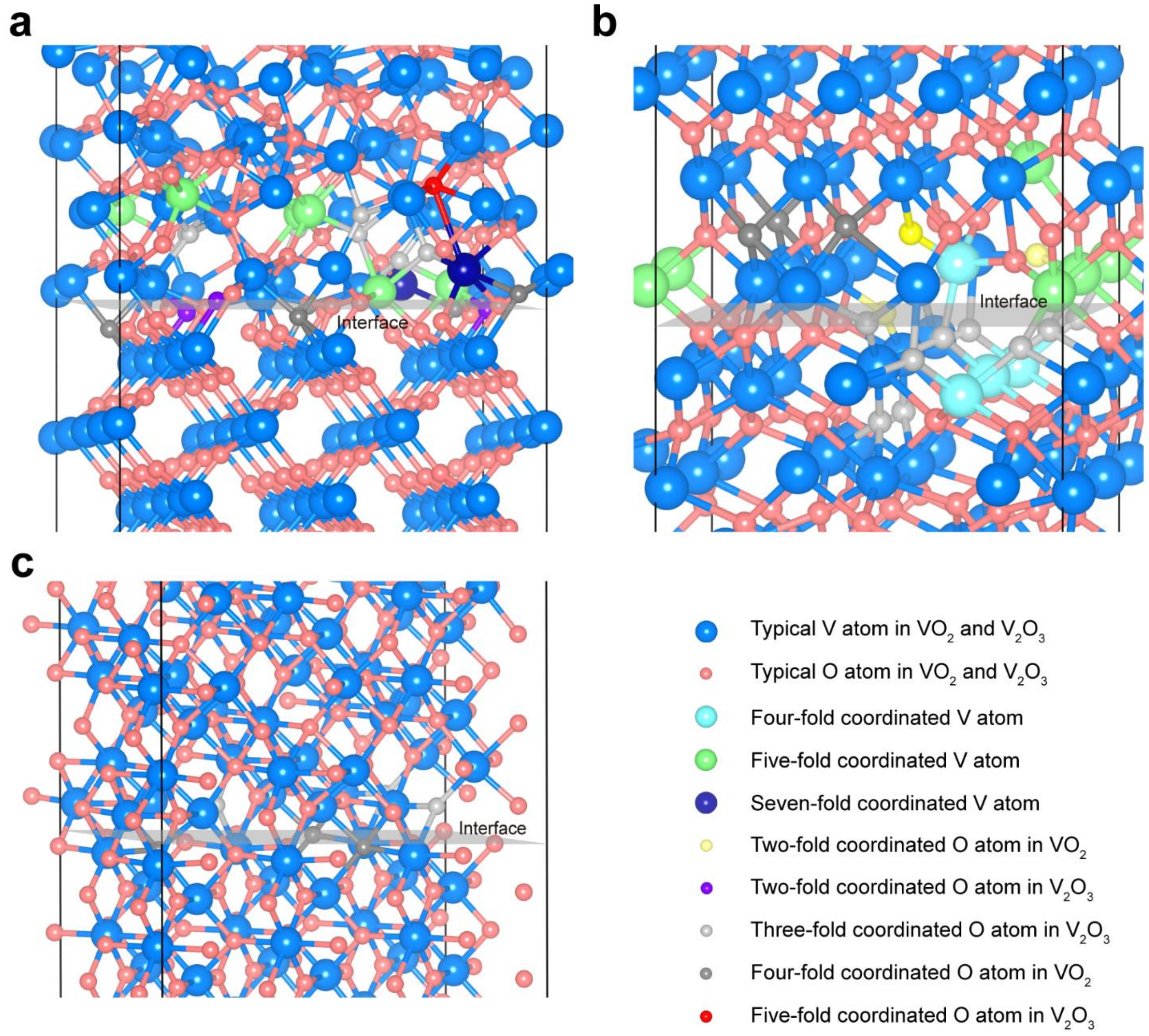

Figure S18 Local chemical environments for both V and O ions near the interface region for (**a**) interface I, (**b**) interface II and (**c**) interface III, respectively.

Table S1. Lattice mismatch between VO_2 and V_2O_3 for three constructed interface configurations.

I	13.99 (V_2O_3[0001])	13.59 (VO_2[010]$_M$)	2.9%	8.559 (V_2O_3[-1100])	11.53 (VO_2[100]$_M$)	-25.7%
II	9.884 (V_2O_3[11-200])	9.083 (VO_2[102]$_M$)	8.8%	8.559 (V_2O_3[-1100])	11.53 (VO_2[100]$_M$)	-25.7%
III	13.99 (V_2O_3[0001])	13.60 (VO_2[010]$_M$)	2.9%	9.884 (V_2O_3[11-20])	9.083 (VO_2[102]$_M$)	8.8%

Table S2. Calculated bond deformation across three constructed interfaces. The long and short V-O bonds are the bond out-of- and in- the planar of V-O octahedra. Reference is the bond length in the bulk.

Interface	Bond type	Average (Å)	Reference (Å)
I	long V-O bond	2.013	1.99 (VO_2)
	short V-O bond	1.920	1.95 (VO_2)
II	long V-O bond	2.137	1.99 (VO_2)
	short V-O bond	1.907	1.95 (VO_2)
III	in-plane V-O bond in V_2O_3	1.966	1.97 (V_2O_3)
	in-plane V-O bond in VO_2	1.987	1.95 (VO_2)

Table S3. Count of atoms near the interface that have different coordination (co.) from the bulk.

Interface	four-fold co. V in VO_2	five-fold co. V in VO_2	four-fold co. O in VO_2	two-fold co. O in VO_2	seven-fold co. V in V_2O_3	five-fold co. V in V_2O_3	three-fold co. O in V_2O_3	five-fold co. O in V_2O_3	Total
I	0	0	4	2	1	5	6	1	19
II	3	1	3	3	3	2	7	0	22
III	0	0	3	0	0	0	3	0	6
