# Supplementary material for: Artificially controlled nanoscale chemical reduction in VO2 through electron beam illumination
Source: Nat Commun. 2023 Jul 7;14:4012. doi: 10.1038/s41467-023-39812-8 (PMC10329014; doi:10.1038/s41467-023-39812-8)
Supplement: Supplementary file 3 — Reporting Summary [file 41467_2023_39812_MOESM3_ESM.pdf]

nature research

Corresponding author(s): Pu Yu

Lasing Reporting Summary

Nature Research wishes to improve the reproducibility of the work that we publish. This form is intended for publication with all accepted papers reporting claims of lasing and provides structure for consistency and transparency in reporting. Some list items might not apply to an individual manuscript, but all fields must be completed for clarity.

For further information on Nature Research policies, including our data availability policy, see Authors & Referees.

ü Experimental design

Please check: are the following details reported in the manuscript?

1. Threshold

Plots of device output power versus pump power over a wide range of values indicating a clear threshold
- [ ] Yes
- [x] No

This work is not related to lasers build on new materials.

2. Linewidth narrowing

Plots of spectral power density for the emission at pump powers below, around, and above the lasing threshold, indicating a clear linewidth narrowing at threshold
- [ ] Yes
- [x] No

This work is not related to lasers build on new materials.

Resolution of the spectrometer used to make spectral measurements
- [ ] Yes
- [x] No

This work is not related to lasers build on new materials.

3. Coherent emission

Measurements of the coherence and/or polarization of the emission
- [ ] Yes
- [x] No

This work is not related to lasers build on new materials.

4. Beam spatial profile

Image and/or measurement of the spatial shape and profile of the emission, showing a well-defined beam above threshold
- [ ] Yes
- [x] No

This work is not related to lasers build on new materials.

5. Operating conditions

Description of the laser and pumping conditions
Continuous-wave, pulsed, temperature of operation
- [ ] Yes
- [x] No

This work is not related to lasers build on new materials.

Threshold values provided as density values (e.g. W cm^{-2} or J cm^{-2}) taking into account the area of the device
- [ ] Yes
- [x] No

This work is not related to lasers build on new materials.

6. Alternative explanations

Reasoning as to why alternative explanations have been ruled out as responsible for the emission characteristics
e.g. amplified spontaneous, directional scattering; modification of fluorescence spectrum by the cavity
- [ ] Yes
- [x] No

This work is not related to lasers build on new materials.

7. Theoretical analysis

Theoretical analysis that ensures that the experimental values measured are realistic and reasonable
e.g. laser threshold, linewidth, cavity gain-loss, efficiency
- [ ] Yes
- [x] No

This work is not related to lasers build on new materials.

8. Statistics

Number of devices fabricated and tested
- [ ] Yes
- [x] No

This work is not related to lasers build on new materials.

Statistical analysis of the device performance and lifetime (time to failure)
- [ ] Yes
- [x] No

This work is not related to lasers build on new materials.
